# Treatment of Anemia in Inflammatory Bowel Disease– Systematic Review and Meta-Analysis

**DOI:** 10.1371/journal.pone.0075540

**Published:** 2013-12-02

**Authors:** Tomer Avni, Amir Bieber, Tali Steinmetz, Leonard Leibovici, Anat Gafter-Gvili

**Affiliations:** Medicine E, Rabin Medical Center, Beilinson Hospital, Petah-Tikva, and the Sackler School of Medicine, Tel-Aviv University, Tel-Aviv, Israel; The University of Colorado, United States of America

## Abstract

**Background:**

Anemia is considered the most common systemic complication of inflammatory bowel disease (IBD). We aimed to provide all available evidence regarding the safety and efficacy of therapy existing today to correct anemia in IBD.

**Methods:**

Systematic review and meta-analysis of randomized controlled trials that compared any treatment for anemia in patients with IBD. We searched electronic databases, conference proceedings and clinical trials registries. Two reviewers independently extracted data from included trials. The primary outcome was the effect of treatment for anemia in IBD on the hemoglobin (Hb) response, defined as rate of patients who achieved an increase of 2 g/dl in Hb concentration at the end of the follow-up. Secondary outcomes included disease severity scores, iron indices, Hb levels, inflammatory markers, adverse effects, and mortality. Dichotomous data were analysed by calculating the relative risk (RR) for each trial with the uncertainty in each result being expressed using 95% confidence intervals (CI). A fixed effect model was used, except in the event of significant heterogeneity between the trials (P<0.10, I^2^>40%), in which we used a random effects model.

**Results:**

Nine trials fulfilled the inclusion criteria, to a total of 973 patients. We were able to perform meta-analysis for intravenous (IV) versus oral iron and for ESAs versus placebo. IV iron was associated with a higher rate of achieving Hb response in comparison to oral iron; RR 1.25 (95% CI 1.04–1.51, I^2^ = 2%, 4 trials), CRP levels and disease activity indexes were not significantly affected by IV iron. IV iron was associated with a decrease in adverse events that required discontinuation of intervention and without an increase in serious adverse.

**Discussion:**

Treatment for anemia in IBD should include IV iron and not oral iron replacement, due to improved Hb response, no added toxicity and no negative effect on disease activity.

## Introduction

Anemia is considered the most common systemic complication of inflammatory bowel disease (IBD) [1], [2]. It negatively affects quality of life (QOL), cognitive function, functional status [3], [4], and is a co-morbid condition that is associated with other diseases (e.g. transfusion- associated hepatitis C) or even death [5]. Prevalence of anemia in IBD patients varies from as low as 6% [6] and as high as 74% [7] in different cohorts.

Anemia in IBD has multiple causes with iron deficiency anemia, anemia of chronic disease and a combination of both [2], being the most prevalent [8]. Almost every anemic patient with IBD demonstrates some degree of iron deficiency, and the prevalence varies between 36% and 90% [9]. Other less common causes for anemia in IBD include megaloblastic anemia with a prevalence up to 26.6% [10], of Crohn's disease patients, drugs as sulfasalazine [11], purine antagonists [12], Interleukin-10 antagonists [13] and autoimmune hemolytic anemia [14]. Several review studies have addressed the epidemiologic, etiologic, or therapeutic aspects anemia in IBD [15]–[17].

Due to the diversity of etiologies for anemia, several treatment options were studied. Treatment options contain oral and intravenous (IV) iron preparations, erythropoiesis stimulating agents (ESA) supplemented with iron, and red blood cell transfusions.

We therefore performed this systematic review and meta-analysis assembling the current data from randomized controlled trials in order to provide the highest quality of evidence regarding the safety and efficacy of therapy existing today to correct anemia in IBD.

## Methods

Systematic review and meta-analysis of randomized controlled trials.

### Study Selection

We included all randomized controlled trials comparing any treatment for anemia in patients with IBD. Anemia was defined in each trial by the authors. All available therapies were included with any comparison between oral or IV iron preparations, ESAs, red blood cell transfusions and placebo. We included trials regardless of publication status (published, conference proceedings, or unpublished), trial years, and language.

### Data sources

We searched MEDLINE (1/1966 to 1/2013), the Cochrane Central Register of Controlled Trials (CENTRAL) (The Cochrane Library, Issue 3 of March, 2013 and NLM gateway. We also searched conference proceedings of the American Society of Gastroenterology, from 2007 onwards. We also searched clinical trials databases for ongoing and unpublished trials: http://www.controlled-trials.com, http://www.clinicaltrials.gov/ct and http://clinicaltrials.nci.nih.gov. The references of all identified studies were inspected for more trials. The terms “inflammatory bowel disease OR Crohn's disease OR ulcerative colitis,” were searched as both medical subjects heading terms (MeSH) and as text words and crossed with “iron” (MeSH and a text word) and specific iron preparations; “erythropoietin stimulating agents” and specific ESA preparations and “anemia”. The result was limited to randomized controlled trials using a highly sensitive filter [18]. We did not identify any controlled trial of red blood cell transfusions or vitamins supplementation.

### Data Extraction and Quality Assessment

Two reviewers independently extracted data from included trials. In case of disagreement between the 2 reviewers, a third reviewer extracted the data and results were attained by consensus. We contacted the investigators of included trials for missing data.

We assessed trials for methodological quality and examined the following domains: random sequence generation, allocation concealment, blinding of participants and personnel, blinding of outcome assessment, incomplete outcome data reporting, selective outcome reporting. We graded each domain as low risk for bias, unclear risk -lack of information or uncertainty over the potential for bias, or high risk for bias according to the criteria specified in the Cochrane Handbook version 5.1.0 (*Cochrane Handbook for Systematic Reviews of Interventions* Version 5.1.0 [updated March 2011] [18], [19].

### Definition of Outcomes

The primary outcome we extracted was the effect of treatment for anemia in IBD on the hemoglobin response, which was defined as the rate of patients who achieved an increase of 2 g/dl in hemoglobin (Hb) concentration at the end of the follow-up. Secondary outcomes included disease severity scores (Inflammatory Bowel Disease Questionnaire (IBDQ) scores [20], The Harvey-Bradshaw Simple Index scores (HBSI) [21], Crohn's Disease Activity Index (CDAI) diary card [22] and UC [23]); iron indices (ferritin concentration and transferrin saturation (TSAT), Hb levels or absolute change in Hb level at the end of follow-up; red blood cell transfusion requirements, inflammatory markers (CRP levels); number of patients with treatment failure; adverse effects (AEs) (severe AEs, AEs leading to discontinuation and by involved organ), QOL scores, and mortality.

### Data Synthesis and Analysis

Dichotomous data were analysed by calculating the relative risk (RR) for each trial with the uncertainty in each result being expressed using 95% confidence intervals (CI).We obtained mean and SD values for continuous variables. When mean or SD values were not available, we calculated them by using data obtained from figures or by recalculating them from other effect estimates and dispersion measures [18] (In one trial [24], we were unable to determine SD for HB and ferritin levels from publication and authors, therefore data from similar trial [25] was used instead as their methodology and setting were close [26]). For continuous variables we calculated weighted mean difference (WMD) for variables that were reported on the same scale. WMD represents the weighted combination of absolute differences between the mean values in the two groups in a clinical trial. For continuous data reported in different scales (for example different disease activity scales) we used the standardized mean difference (SMD). Alternatively, to allow pooling of results from patients with CD and UC, disease activity scores were calculated as actual score divided by maximum score. Heterogeneity (degree of difference between the results of different trials) was assessed by calculating Chi-square and I^2^ tests of heterogeneity. A fixed effect model was used throughout the review, except in the event of significant heterogeneity between the trials (P<0.10, I^2^>40%), in which we used a random effects model. Data from cross-over trials was used only if reported at the time of the first cross-over and separately for each arm of intervention. We conducted several comparisons and results were pooled and stratified by intervention type: oral versus IV iron, subcutaneous ESAs versus placebo, different oral preparations, different IV iron preparations and IV iron versus placebo. Due to the paucity of data we were unable to further stratify results by type of iron and ESAs preparation, age or gender and underlying IBD.

## Results

**Figure 1:**
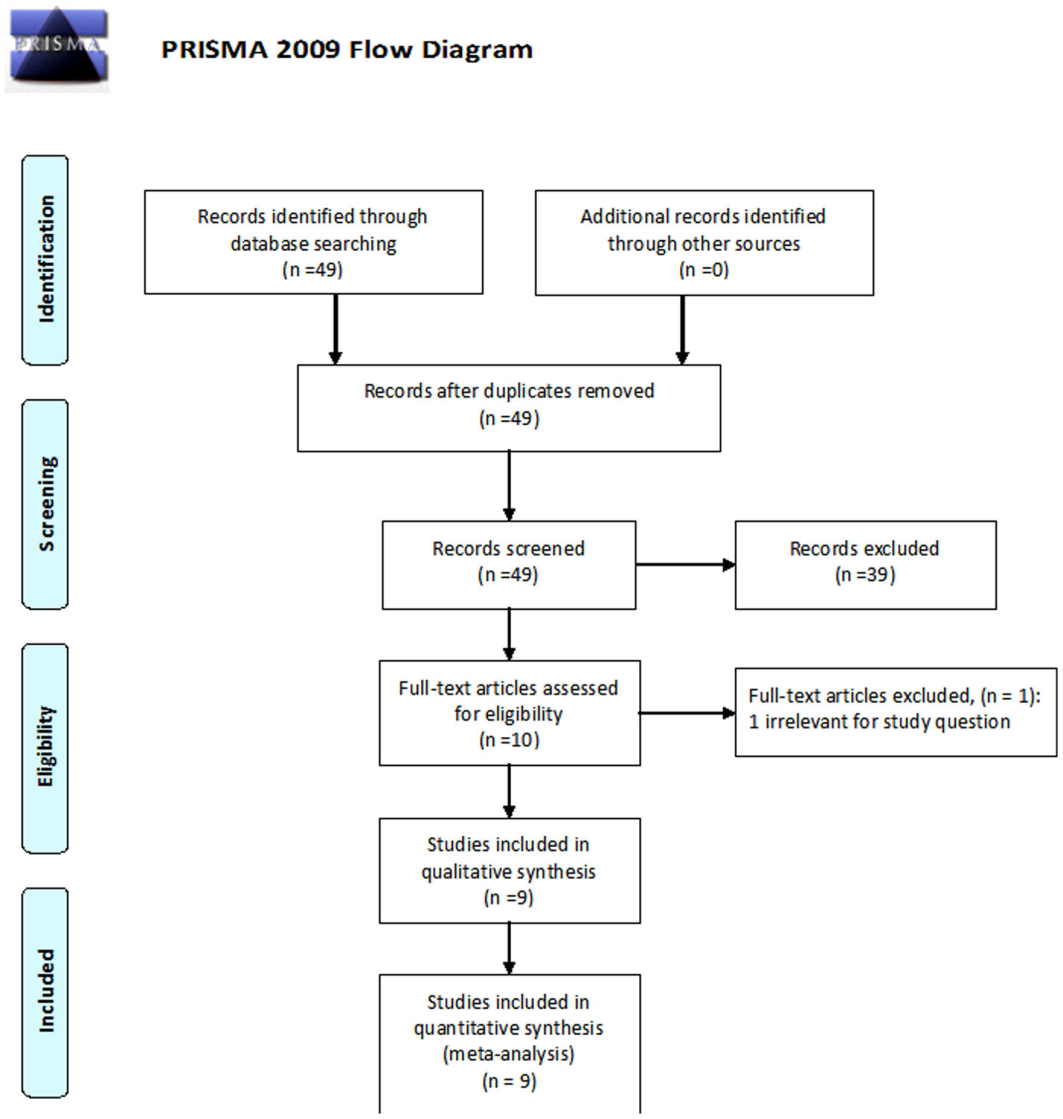
PRISMA flow diagram.

The literature search identified 48 publications; of them, 9 were potentially eligible publications on anemia therapy in patients with IBD. Nine trials [24], [25], [27]–[33] performed between 1996 and 2013 fulfilled the inclusion criteria (studies flow chart, figure 1). Four trials compared oral to IV iron, 1 trial compared two oral iron preparations, 1 trial compared two IV iron preparations and 1 trial compared IV iron to placebo; 2 trials compared ESAs to placebo. We were able to perform meta-analysis for IV versus oral iron and for ESAs versus placebo, and results are presented separately. A total of 973 patients were recruited, of them 395 (40.5%) suffering from Crohn's disease and 578 (59.5%) from ulcerative colitis. Most patients were young adults (median age ranged from 26–46) and females (60.8%). All trials included patients with anemia, although the definition of anemia varied considerably between the trials (Table 1). Concurrent medication at enrolment included 5- aminosalysilic compounds (used by 53% of patients), systemic steroids (24.2%), azathioprine or 6 mercaptopurine (14.3%) and anti TNFs (4.5%). Disease severity indexes at enrolment varied considerably between studies (median CAI between 1–11, median HBSI 2–4, median CDAI 84–281, Table 1).

**Table 1:** Studies characteristics.

Study	Drug and dosage	Maximal planned dosage of iron	Number of Patients randomized	Follow up duration	Haematological Inclusion Criteria: TSAT (%)/Ferritin (ng/mL)/Hb (g/dL)	Mean Hb (g/dL) at enrolment	% Female	Mean age	% patients with CD/UC	Disease severity score (CAI, HBSI, CDAI)
Erichsen 2005a [28]	IV iron sucrose 200 mg, 3 times over 2 weeks	600 mg	17	2 weeks	NS, NS, <12/13	10.6–11.6	68	18–46 (range)	57/43	med 1 (0–3)/1 (0–4), med 3.5 (1–7)/4 (2–5), NS
	Oral Ferrous fumarate, 120 mg daily, 2 weeks	1,680 mg								
Erichsen 2005b [27]	Oral ferrous sulphate, 200 mg daily, 2 weeks	2,800 mg	21	2 weeks	NS, <15, NS	13.1	61	41	62/38	med 1 (0–7), med 3 (0–8), NS
	Oral Maltofer Film tablets, 200 mg daily, 2 weeks	2,800 mg	20			12.5	60	31	55/45	med 2 (0–5), med 2 (0–10), NS
Evstatiev 2011 [29]	IV Ferric carboxymaltose 500–1,000 mg, once weekly, 3 weeks	3,000 mg	244	12 weeks	NS, >100, 7–12/13	10.1	60	39.5	35/65	m 3.7 (2.1), NS, m 97.5 (61.2)
	IV iron sucrose 200 mg, 1–2 times weekly, 3 weeks	2,200 mg	239			10.3	58	38	31/69	m 3.2 (2.2), NS, m 84.5 (61.7)
Gasche 1997 [30]	Erythropoietin alfa 150 u/kg, thrice weekly, 8–16 weeks	3,200 mg, IV	20	16 weeks	NS, NS, <10.5	8.7	85	32	100/0	NS, NS, NS
	placebo	3,200 mg, IV	20			8.5	50	32	100/0	NS, NS, NS
Kulnigg 2008 [25]	IV Ferric carboxymaltose 500–1,000 mg, once every 4 weeks, 12 weeks	3,000 mg IV	136	12 weeks	<20%, <100, <11	8.7	59	40	30/70	med 8 (0–14), NS, med 217 (72–424)
	Oral ferrous sulphate, 200 mg daily, 12 weeks	16,800 mg PO	60			9.1	60	45	26/74	med 7 (0–15), NS, med 238 (63–363)
Kulnigg-Dabsch 2013 [31]	IV Ferric carboxymaltose 500 mg, once every 2 weeks, 3 weeks	1,500 mg IV	16	6 weeks	<20%, <100, >10.5	11.7	75	36.1	68/32	m 5 (2), NS, med 168 (99–229)
	placebo	NA	9			11.1	88	31.6	44/56	m 4 (1), NS, med 187 (116–207)
Lindgren 2009 [24]	IV iron sucrose 200 mg, once every 1–2 weeks, 20 weeks	8,000 mg IV	45	20 weeks	NS, <300, <11.5	10.5	71	42	44/56	med 3.4 (0–10), med 3.9 (0–12), NS
	Oral ferrous sulphate, 400 mg daily, 20 weeks	56,000 mg PO	46			10.4	67	42	52/48	med 3.1 (0–8), med 3.2 (0–8), NS
Schreiber 1996 [32]	Erythropoietin alfa 150 u/kg, twice weekly, 12 weeks	12,600 mg, PO	17	12 weeks	NS, >20/30, NS	8.8	17	26	53/47	med 5.5 (3–10), NS, med 187 (135–312)
	placebo	12,600 mg, PO	17			8.6	35	31	42/58	med 6 (4–11), NS, med 213 (141–412)
Schr¨oder 2005 [33]	IV iron sucrose 200 mg, 1–2 per week, 5 weeks	2,500 mg IV	22	6 weeks	<20%, <20, <10.5/11	9.8	77	35	77 23	med 11 (7–19), NS, med 217 (46–417)
	Oral ferrous sulphate, 100–200 mg daily, 5 weeks		24			9.6	70	33	50/50	med 8 (4–11), NS, med 281 (71–423)

Data presented in table in mean (m) with (standard deviation) or median (med) with (range), as presented in original studies.

Abbreviations: CD-Crohn's disease; Hb - hemoglobin; IV -intravenous; NS - not specified; PO – per os; TSAT - transferrin saturation; UC - ulcerative colitis; CDAI - Crohn's Disease Activity Index, HBSI - The Harvey-Bradshaw Simple Index, CAI – Colitis activity index.

### Risk of bias assessment

**Figure 2:**
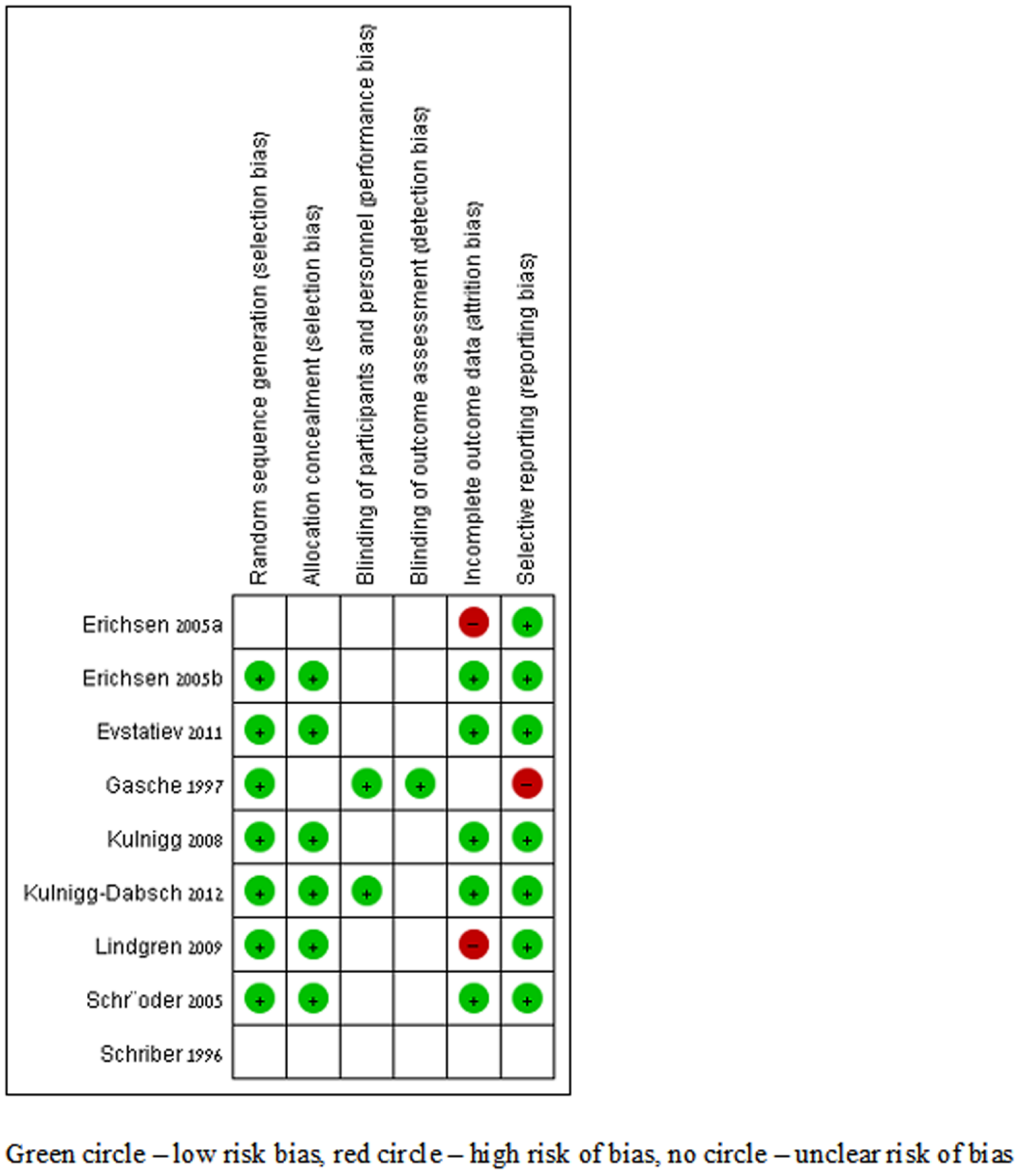
Risk of bias assessment.

Figure 2 summaries results for risk of bias. Allocation generation was adequate (low risk for bias) in 6 trials; allocation concealment was adequate in 5. One trial was double blinded [30]. In 5 trials, the primary outcome was analysed by intention to treat.

### IV versus oral iron

Planned total IV iron dosages ranged between 1,000 mg to 2,000 mg. Reported administered dosages ranged from 980 mg to 1,700 mg. Patients were followed up between 6–20 weeks, without reported losses to follow up. All patients suffered from anemia at enrolment, however, due to different inclusion criteria, in the trial by Erichsen et al. [28] patients had a higher baseline Hb level and a higher TSAT. Ferritin levels were remarkably low at enrolment (median values ranged from 5.0 to 19 µg/L). Patients in studies by Erichsen et al. [28] and Lindgren et al. [24] had more quiescent disease at enrolment in comparison to studies by Schroder et al. [33] and Kulnigg et al. [25] as presented by higher CAI, CDAI and HBSI scores (Table 1).

#### Primary Outcome

**Figure 3:**
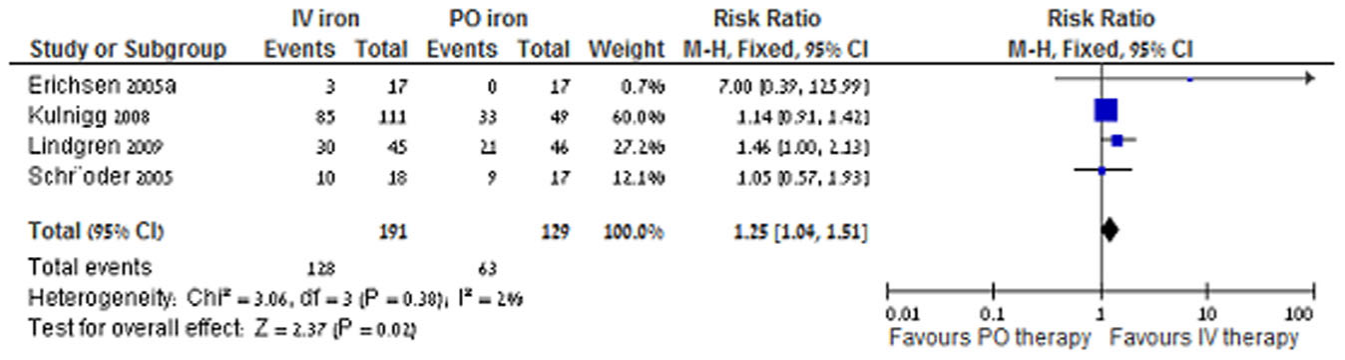
IV iron versus PO iron, Hb response at end of follow-up.

IV iron was associated with a higher rate of achieving a 2 g/dl increase in Hb concentration in comparison to oral iron; RR 1.25 (95% CI 1.04–1.51, I^2^ = 2%, 4 trials, Figure 3), and a risk difference of 0.13 (number needed to treat 7.69). A sensitivity analysis according to methodological quality excluding the one trial with unclear risk for bias [28] showed similar results (RR 1.21 95% CI 1.01–1.46, I^2^ = 0%, 3 trials).

#### Iron indices

Ferritin levels were significantly elevated with IV iron treatment in comparison to oral iron preparation by a WMD of 107.5 ng/mL (95% CI 24.7–190.2, I^2^ = 99%, random effects model), however, the trials were significantly heterogeneous with no single study accounting for it. TSAT was not significantly affected with either intervention achieving an absolute decrease of WMD 0.91% (95% CI −7.87–6.05, I^2^ = 97%, random effects model) with IV iron. The IV route was associated with a greater improvement in the Hb level than the oral route, WMD in Hb of 0.2 g/L (95% CI 0.02–0.39, I^2^ = 95%, random effects model).

#### Clinical parameters

CRP levels were not significantly affected by IV iron (WMD 0.35 mg/l, 95% CI-1.51–2.42, I^2^ = 75%, random effects model). Disease activity indexes were also not significantly affected by IV iron (for UC, CAI score, WMD of 0.45 points, 95% CI 0.82–1.71, I^2^ = 88%, random effects model; for CD, combined score of CDAI and Harvey-Bradshaw Simple Index score WMD 0.95 95% CI −2.51–4.4, I^2^ = 0%). There were no data in the trials regarding QOL scores or all-cause mortality.

#### Adverse events

**Figure 4:**
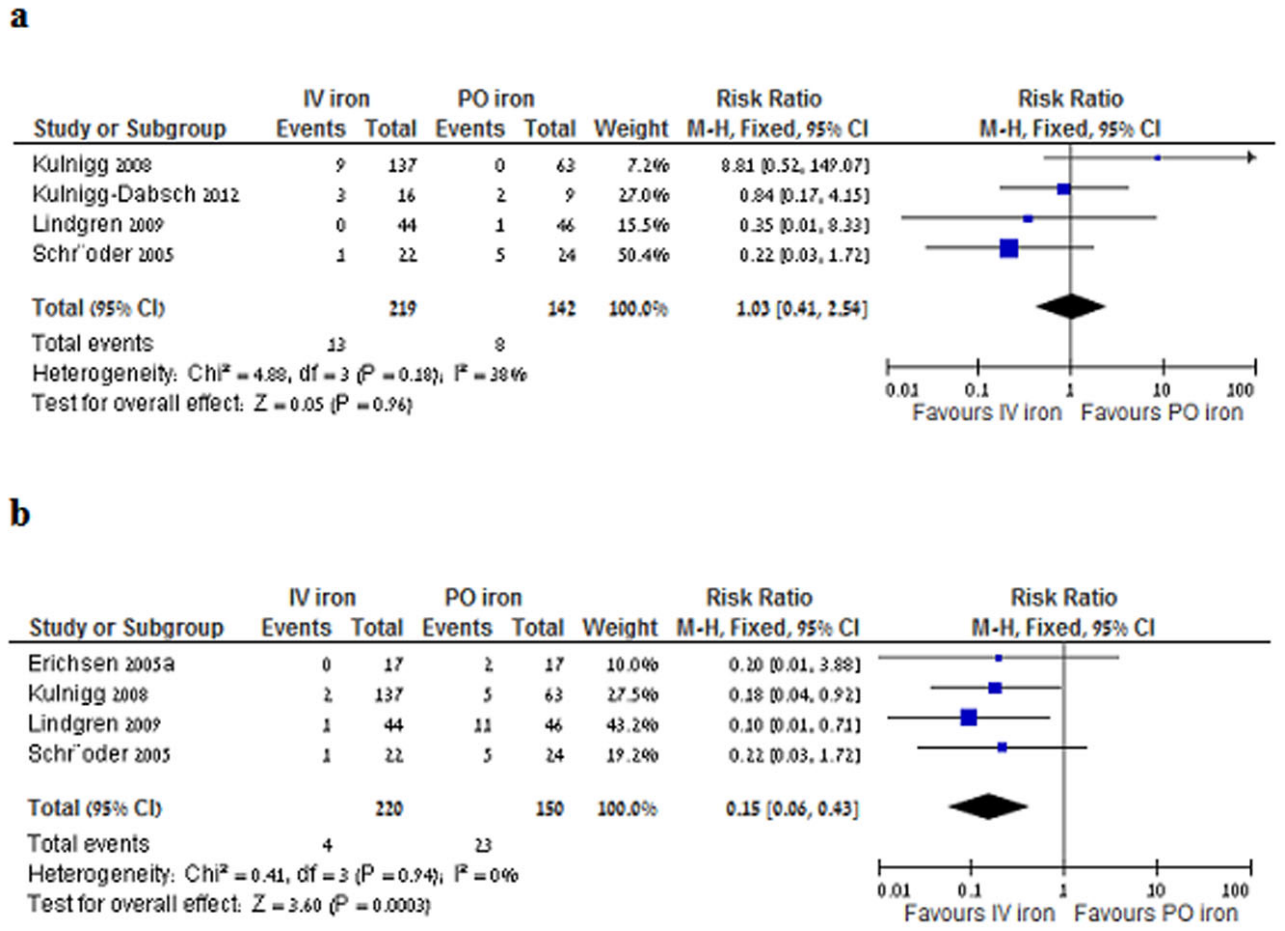
A IV iron versus PO iron, serious AEs; b AEs requiring discontinuation.

All studies reported on the presence or absence of AEs. Safety data from Kulnigg-Dabsch et al. [31] was added to the analysis to assess IV iron preparations as a group. There was no increase in serious AEs (defined as anaphylaxis, adverse events requiring hospitalization, and adverse events regarded by authors as serious) with IV iron (RR1.03 95% CI 0.4–2.6. I^2^ = 41%, figure 4a); IV iron was associated with a decrease in AEs that required discontinuation of intervention (RR 0.13, 95% CI 0.05–0.38, I^2^ = 0%, figure 4b); decrease in gastrointestinal AEs (abdominal pain, diarrhea, vomiting, flatulence and bleeding) (RR 0.25, 95% CI 0.06–0.95, I^2^ = 61%, random effects model); However, there was an increase in non-serious transfusion reaction (rash, urticaria, rigors, tachycardia, peripheral edema) (RR 3.07, 95% CI 1.23–7.6, I^2^ = 30%).

### IV FCM versus IV iron sucrose

One trial [29] compared FCM directly to iron sucrose. Follow up time was 12 weeks. FCM was associated with a higher rate of achieving a 2 g/dl increase in Hb concentration in comparison to iron sucrose by a RR of 1.65 (95% CI 1.11–2.38). Improvement in QOL scores and disease severity scores were not different between the study arms. The occurrence of serious AEs was not different between interventions.

### IV FCM versus placebo

One study [31] compared FCM to placebo. There were no data regarding Hb response. Iron indices (ferritin, TSAT) and Hb level improved significantly with FCM but not with placebo. QOL and disease severity scores improvement and the occurrence of serious AEs were similarly improved with both interventions without statistically significant advantage in either treatment.

### Oral iron sulphate versus oral iron–polymaltose complex

One trial [27] compared between two oral iron preparations. There were no data regarding Hb response. CRP levels significantly decreased with iron sulphate WMD −5.0 mg/L (95% CI −6.22–3.78). Iron sulphate was also associated with improved iron indices (ferritin levels increased by WMD 12, 95% CI 10.45–13.55; TSAT increased by WMD 5.3% 95% CI 4.15–6.45). In addition, Hb levels increased by a WMD of 0.8 g/L, 95% CI 0.62–0.98). A non-statistically significant increase in gastrointestinal AEs and AEs requiring discontinuation of intervention occurred with iron sulphate.

### ESAs therapy - ESAs versus placebo

ESAs were studied in 2 trials versus placebo [30], [32]. Both trials used epoetin alfa supplemented with IV iron replacement therapy in the intervention arm. Follow-up duration ranged between 12–16 weeks. Planned administered epoetin alfa dose was between 3,200–12.600 IUs (Table 1).

#### Primary Outcome

ESA administration was associated with a non-significant increase in the rate of achieving a 2 g/dl increase in HB levels, RR 1.99 (95% CI 0.56–7.14, I^2^ = 87%, random effects model).

#### Secondary Outcomes

No data were available for clinical outcomes for these trials.

#### Iron indices and Hb level

Hb levels increased with ESA by a WMD of 2.32 g/L (95% CI 1.33–3.32, I^2^ = 63%). Iron indices were not reported.

#### Adverse events

Gasche et al. [30] reported an increased rate of transfusion reactions to ESAs, although it was statistically insignificant, RR 3.21 (95% CI 0.54–19.11).

## Discussion

In this systematic review and meta-analysis we examined the current evidence for the treatment of anemia in IBD patients. We conducted several comparisons, the important one being the comparison of IV iron to oral iron. The important findings of our meta-analysis include the better Hb response achieved with IV iron preparations compared to oral iron, with an acceptable safety profile and higher rates of adherence or reduced discontinuation of intervention in the IV iron arm.

We also showed a significant increase in ferritin level and Hb value (as a continuous variable) with IV iron therapy. Moreover, disease activity indexes were not negatively influenced by IV iron. CRP values and QOL scores were unaffected by either preparations (although reporting methods and measurement scales varied considerably between studies). There were not enough data to consider further analyses of Hb response according to disease type, presence of any anemia or iron deficiency anemia at enrollment, type of iron preparation and methodological sensitivity analyses.

As for the optimal IV iron preparation, the comparison of IV versus oral iron included 3 trials of iron sucrose and one of FCM, all showing similar results. Only one trial compared between iron sucrose and FCM [29] and FCM proved to be more efficacious than iron sucrose in achieving hemoglobin response. FCM is more convenient to patients because which usually requires only one to two infusions (up to 1 g per dose), while iron sucrose usually requires five to 10 infusions to reach total dose because 200 mg is considered the well tolerated individual dose. In a previous meta-analysis, FCM was demonstrated to be superior to iron sucrose and oral iron in achieving an increased HB level with a similar rate of serious AEs and mortality [34]

We also showed a trend towards a better Hb response with the use of ESA (supplemented by IV iron), without the occurrence of serious adverse events. This is probably since the anemia in IBD is multi-factorial, and anemia of chronic disease is one of the mechanisms. ESA has been shown to be efficient in achieving hemoglobin response in other situations of anemia of chronic disease as chronic kidney disease and cancer related anemia [35], [36].

Although the studies had very different interventions, inclusion and exclusion criteria and follow-up time, the clinical outcomes (Hb response, disease severity scores and AEs) had low heterogeneity across all analyses, supporting the robustness of our findings. We did encounter high heterogeneity when we pooled iron indices results, probably due to the different baseline iron measurements across the studies.

Prior reviews had examined therapy for anemia in IBD patients. In 2006, a systematic review by Kulnigg and Gasche [15] examined all available trials present. The authors examined both observational and interventional studies and also animal models. The authors concluded that disease activity may determine the severity of anemia in CD, and the role of iron supplementation (IV and oral) should be further explored in clinical trials. Recently, a meta-analysis by Lee et al. [37] had examined the role iron therapy for IBD, by comparing oral to IV therapy and concluded, similarly to our findings, that IV iron is superior to oral therapy in achieving an increase in Hb value and reduced risk of discontinuation. Our review adds to the Lee review, by showing both an increase in the rate of patients achieving a hemoglobin response and in the Hb value, thus analyzing the primary outcome of Hb response as a clinical event, rather by using Hb value as a continuous variable. In addition we included more trials, with triple the number of patients, and included comparisons of all interventions for anemia available for analysis (e.g. inclusion of ESAs and comparison of different oral and iron preparations.

The advantages of IV iron over oral therapy have been demonstrated previously in other clinical settings. IV iron was proven to be superior to oral iron replacement in achieving Hb response, in patients suffering from chronic kidney disease and especially when on dialysis [38]. IV iron added to ESA resulted in an increase in Hb response and a reduction in the need for red blood cell transfusions in patients with chemotherapy-induced anemia [39]. IV iron therapy was also associated with improved quality of life parameters, reduction in hospitalizations, and increased exercise performance in patients with symptomatic chronic heart failure [40]. No increased risk of serious adverse events was found in these meta-analyses. ESAs have also been shown to be effective in improving Hb levels and quality of life in several chronic conditions (dialysis [35], chronic heart failure [36], cancer [41], rheumatoid arthritis [42]), although an increased risk for thromboembolism and mortality was found in some studies [43].

In 2007, an international working party developed guidelines for evaluation and treatment of anemia and iron deficiency in IBD patients [44]. The working party agreed upon 16 guidelines regarding diagnostic measures to screen for iron- and other anemia-related deficiencies regarding the triggers for medical intervention, treatment goals, and appropriate therapies. Each guideline was graded on the category of the evidence supporting it [45]. Evidence for treatment with iron supplements for iron deficient and anemic patients, and with ESA for ACD was graded as grade A and grade B recommendations, respectively. The preferred route of iron supplementation in IBD according to these guidelines is IV (Grade A). For ESA, treatment should be combined with IV iron supplementation (grade A) [46], [47]. Our results are in agreement with these 2007 guidelines. Our findings strengthen the recommendation of IV iron as the preferred route according to the better hemoglobin response achieved. Although we did not prove a change in QOL and disease severity score with the use of IV iron, and we lacked data regarding QOL in other interventions, there is some evidence that all iron interventions decrease the disease severity score.

The strengths of this review are its comprehensiveness, meaning, the inclusion of all available studies and interventions, inclusion of unpublished trials, the use of a dichotomous variable for Hb response (rate of patients who achieved it) rather than a continuous variable review (which may be more convenient to treating physicians than laboratory values) and solid statistical analysis. Our review has several limitations. First, clinical data was sparse and was presented by authors in different measuring scales, severity indexes and statistical methods. Second, the trials had different follow-up duration ranging from 2 to 20 weeks, however most trials that examined IV iron followed their patients for a similar amount of time (12–20 weeks). Third, Hb values for the definition of anemia (or inclusion criteria) and for the primary outcome of Hb response varied between studies. Fourth, the protocols used for interventions (although similar) did differ between trials. The actual amount of elemental iron delivered for the patients due to the different administration schedules and the bioavailability of the different compounds may also play a role in determining the actual Hb response.

Implications for practice and research: Treatment for anemia in IBD should include iron. The preferable route according to current evidence is IV and not oral iron replacement, due to improved hemoglobin response, no added toxicity and no negative effect on disease activity. ESA therapy may also be used in order to treat the anemia of chronic disease that usually accompanies iron deficiency in IBD. As for the optimal IV iron type and schedule – future trials should further explore the most efficacious administration schedule and dosages by directly comparing between the different iron compounds and schedules. In order to define the role of ESA, future trials should compare IV iron to IV iron with the addition of ESA.

## Supporting Information

Checklist S1 PRISMA Checklist.
(DOC)
